# Levels of Reconstruction as Complementarity in Mixed Methods Research: A Social Theory-Based Conceptual Framework for Integrating Qualitative and Quantitative Research

**DOI:** 10.3390/ijerph7093478

**Published:** 2010-09-16

**Authors:** Linda J. Carroll, J. Peter Rothe

**Affiliations:** 1 Department of Public Health Sciences, School of Public Health, University of Alberta, 3-50 University Terrace, 8303-112 Street, Edmonton, Alberta, T6G 2T4, Canada; 2 Alberta Centre for Injury Control and Research, School of Public Health, University of Alberta, 4075 RTF, 8308-114 Street, Edmonton, Alberta, T6G 2E1, Canada; 3 Centre for Health Promotion Studies, School of Public Health, University of Alberta, 5-10 University Terrace, 8303-112 Street, Edmonton, Alberta, T6G 2T4, Canada; E-Mail: prothe@ualberta.ca

**Keywords:** qualitative research, empirical research, research design, mixed methods research, public health, injury

## Abstract

Like other areas of health research, there has been increasing use of qualitative methods to study public health problems such as injuries and injury prevention. Likewise, the integration of qualitative and quantitative research (mixed-methods) is beginning to assume a more prominent role in public health studies. Likewise, using mixed-methods has great potential for gaining a broad and comprehensive understanding of injuries and their prevention. However, qualitative and quantitative research methods are based on two inherently different paradigms, and their integration requires a conceptual framework that permits the unity of these two methods. We present a theory-driven framework for viewing qualitative and quantitative research, which enables us to integrate them in a conceptually sound and useful manner. This framework has its foundation within the philosophical concept of complementarity, as espoused in the physical and social sciences, and draws on Bergson’s metaphysical work on the ‘ways of knowing’. Through understanding how data are constructed and reconstructed, and the different levels of meaning that can be ascribed to qualitative and quantitative findings, we can use a mixed-methods approach to gain a conceptually sound, holistic knowledge about injury phenomena that will enhance our development of relevant and successful interventions.

## 1. Introduction

The journey from data to knowledge is complex and multifaceted, yet that is the goal of research. This voyage is even more challenging when viewing the world through lenses of different research paradigms, as when both the *Verstehen* (subjective understanding and interpretation, giving rise to qualitative research) and the *positivist* (objective hypothesizing and generalizing, or quantitative research) ways of knowing are considered. This paper describes a way of making the journey from data to a more holistic understanding of phenomena by making the most of the richness of both quantitative and qualitative findings. We present a social-theory-driven approach that conceptualises the integration of quantitative and qualitative research through understanding the different levels of meaning inherent in each. We take a Weber-ian/social constructionist perspective that qualitative and quantitative paradigms form a continuum of reconstructed meaning through *complementarity* [1,2]. We define c*omplementarity* as an epistemological design to understand human behavior through the use of separate but dialectically related research approaches. We believe that, given the impact and complexity of human behavior in matters of health, use of complementarity has special relevance in studying and understanding the wide range of factors related to health and illness in human populations.

Phenomena are experienced. They are interpreted and reproduced through different levels of reconstruction to become ‘data’, the interpretation of which ranges from subjective to objective, ‘common-sense’ to ‘scientific’ [1]. Thus we argue that a meaningful social-theory-based vehicle for mixed methods research is through understanding the dominant research paradigms, their languages and metaphysical assumptions and the interrelationship of ‘inside’ (subjective) and ‘outside’ (objective) observations. One uses a ‘looking in’ perspective, and the other a ‘looking at’ perspective. Using injury research examples, we describe how each paradigm, with its concomitant perspective of ‘looking’ reconstructs the experienced phenomenon to arrive at complementary meanings that are imbued into those phenomenon. Our conceptual framework of complementarity is grounded in epistemology; specifically, that knowledge ranges from practical to theoretical. Each source of knowledge requires a different level of reconstruction of experiences, the combination of which helps us understand the complexity and context of that phenomenon.

## 2. Data, Information and Knowledge

‘Data’ themselves are simply collections of facts or symbols (e.g., numbers or words) with no intrinsic meaning [3]. Data evolve into ‘information’ only when data patterns and relationships are identified and contextualized. ‘Knowledge’ requires yet another level of abstraction, and derives from information. Knowledge is what we hope to achieve through collecting data and doing research.

Data can be quantitative or qualitative, are collected using methods embedded in their own paradigms and are used to address fundamentally different questions. *Quantitative* data answer questions like “how many?” or “how frequently”, and are measured/reported on a numerical scale, permitting categorization of pooled data, numerical reporting, statistical analysis and mathematical modeling. Quantitative data are often considered ‘objective’, although in actuality, the observer’s attitudes about phenomena can affect their measurement.

*Qualitative* data are non-numerical. Qualitative research seeks to analyze verbal discourse through interviews (e.g., interviewing crash survivors), written documents (e.g., newspaper articles reporting crashes), or participatory field observations (e.g., riding with the police to a crash scene). Qualitative data are used to answer questions such as “why?” and “how?” and to capture the *Erleben* or the ‘lived experience’. Although qualitative data can be quantified, we will not address this issue.

*Sources* of data in public health and injury research fall loosely within two broad categories: (a) *primary*, where data are collected specifically for purposes of particular research question, and (b) *secondary*, whereby existing data collected for non-research purposes are used for research. In *quantitative* injury research, examples of primary data sources are structured questionnaires (e.g., surveys on helmet wearing) or researchers’ observations (e.g., counts of bicyclists wearing helmets). Secondary data usually do not involve self-report, although some data sources do (e.g., census data used for research purposes). Secondary data sources such as administrative health databases are frequently used by injury researchers, although their primary purpose is healthcare reimbursement. These sources can yield such information as the frequency of bicycle injuries requiring hospitalization.

Although *qualitative* studies frequently use *primary* data (e.g., interviews), others involve analyses of media reports and other *secondary* data sources. For example, community attitudes about road safety might be explored through interviews (primary data) or by analyzing newspaper articles on rights of bicyclists (secondary data). Each type of research method has its own fundamental assumptions that determine not only how phenomena are studied, but also what aspects of phenomena are studied. These assumptions reflect the researcher’s fundamental epistemological (knowledge, views about the world), ontological (nature of ‘shared reality’) and axiological (values and how they affect actions) stance [4]. Thus, the passage from data to information to knowledge occurs in both paradigms, but in distinct forms that reflect their different fundamental assumptions.

Traditionally, public health research in general, and injury research in particular, has focused mainly on *quantitative* approaches. However, in the last decade or so, there has been growing recognition that qualitative approaches can add to our understanding [5]. It is important to know that a particular group of people engage in risk-taking behaviour (through quantitative research), and also to understand “why?” and “how?” (qualitative research). A more complete ‘knowledge’ requires both ‘objective’ observations, and an understanding of the personal significance and the context within which that injury occurs. For example, quantitative methods have determined the protective effect of booster car seats for children [6] and assessed the role of legislation in increasing booster seat usage [7], while qualitative methods have explored parents’ reasons for their decisions about booster seats [8,9]. Likewise, the implementation of “designated driver” programs to decrease drunk driving has been shown in quantitative studies to have disappointing results [10,11]; qualitative studies explain why and describe dangers faced by designated drivers [12]. Qualitative studies can also explore the *cultural* component of injuries, for example, the social norms and ideology shared by some First Nations communities in Canada, which contribute to risk taking and high injury rates [13].

More recently we have also witnessed an accelerated interest in *mixed methods* in health and public health research, in which studies use both quantitative and qualitative methods [14]. And there are many thoughtful books and papers describing common purposes, methods and analysis strategies for mixed-methods studies [15–22]. However, combining qualitative and quantitative paradigms should not be done without first considering their intrinsic philosophical differences [23]. In fact, some posit that qualitative and quantitative methods are inherently incompatible [24]. At the spectrum’s other end, these two methods have been combined in purely pragmatic ways, e.g., simply using quotes and anecdotes from interviews to buttress the findings of quantitative research. In contrast to both views, we argue that qualitative and quantitative methods and findings *can* be integrated, but only in a conceptually sound way that accounts for their different underlying assumptions about reality and about how reality becomes known and understood [4,25]. Rothe (this paper’s second author) has proposed a social-theory-based conceptual framework for the integration of qualitative and quantitative research that respects the separate fundamental basis for each [4,25–27].

## 3. Framework of Complementarity

Rothe’s framework has its foundation in the philosophical concept of complementarity which has historical roots in the physical sciences.

### 3.1. Complementarity as Rooted in the Physical Sciences

Although physics is considered primarily a physical, objective science, a number of physicists, including Compton, de Broglie, Bohr and Heisenberg, concluded that human influence is integral in interpreting and understanding scientific observations [28–31]. Heisenberg’s formulation of the Uncertainty Principle [31] led to Bohr’s version of complementarity and wave-particle duality [28]. Bohr concluded that depending on the measurement used, mechanical entities reveal either particle-like or wave-like properties—that measurement instruments actually define the conditions under which phenomena appear. Thus, the notions of complementarity and uncertainty dictate that all properties and actions in the physical world are somewhat non-deterministic. This has obvious parallels to the measurement and understanding of social behavior.

### 3.2. Complementarity as Rooted in Social Sciences

Historically, the idea of complementarity also found a solid home in the social sciences. For example, Weber advocated the importance of both ‘rational or objective’ (as in quantitative research) and ‘empathic or subjective’ (as in qualitative research) dimensions for understanding human phenomena [32]. This echoes Cooley’s earlier view that ‘statistical’ knowledge is superficial without ‘empathic’ knowledge [33], and others have advocated that a comprehensive view of human phenomena requires a complementary understanding of different *aspects* of the causes and reasons for those phenomena [34–37]. Likewise, Maslow posited that interpreting human behavior (assessed quantitatively), necessarily requires a complementary understanding of that behavior from the individual’s experiential perspective [38]. The two perspectives are distinct but nonetheless make up a whole. As we show later, this has important relevance for understanding factors related to health, injury and disease.

## 4. Complementarity and Bergson’s Box: ‘Looking in’ and ‘Looking at’

According to the metaphysicist Bergson, there are two ways of knowing something—this can be visualized in the form of a box (‘Bergson’s Box’) [39], One way of knowing an object is from the perspective of the inside—involving entry into the object. The other way is by moving around that object—looking from the outside. In Rothe’s framework, this can be extended to complementarity in investigations of social and individual phenomenon—examining human and social actions from the inside (‘looking in’, or *Verstehen*—qualitative approaches), or examining these actions from the outside (‘looking at’, or *Erklären*—quantitative approaches).

### 4.1. Looking in

Inside the box is subjective meaning—the person’s experiential ‘lived reality’ (also referred to as ‘everyday life’ [1] or *paramount reality*). This reality is *directly* accessible only to that individual, through personal reflection. However, that reality can be examined one step removed when it is relayed to others. Through social experiences—the reciprocal relaying of one’s experiential reality with others (‘looking in’ to each other’s paramount reality), individuals can understand others and be understood. Thus, within the context of the everyday world, individuals gain knowledge through inter-subjectivity, on the fundamental basis that others have similar consciousness, desires and emotions. Persons use their own experiences to understand each other and rely on their experiences with others to understand their own experiences [40,41]. Thus, qualitative researchers may ‘look in’ by asking participants to relay—as fully as possible—their own experienced reality, as would be the case in interviewing community members about their own experiences and attitudes regarding bicyclists and road safety. Researchers then use the participants’ argot and historical/cultural frames of reference to seek to understand those personal experiences; and, across participants, to identify shared streams of consciousness within these experiences. These streams of consciousness represent the paramount reality, or in more empirical terms, they form the categories of meaning that are typically transformed into dominant themes. *Verstehen* requires some degree of reconstruction of the individual’s paramount reality; however the themes closely mirror the participants’ versions of reality. When qualitative researchers use secondary data (e.g., newspaper articles) to explore community attitudes about road safety, the researcher is still ‘looking in’, but at one step removed from direct interaction with community members, thus requiring a different degree of reconstruction of the paramount reality.

### 4.2. Looking at

Whereas knowledge that arises from ‘inside the box’ involves subjective meaning (‘looking in’), the knowledge that arises through examining the ‘outside’ of the box (‘looking at’) represents objective meaning. Here, the quantitative researcher aims at providing generalizable answers, for example, identifying risk factors for a particular injury. This relies on structured, parsimonious language and seeks to classify characteristics and experiences to permit quantification, thus permitting statistical analyses. The analysis results are like photographs of a broken leg taken from various angles; providing important structural details, but not providing insight into trying to walk on that leg.

One way of numerically describing social phenomena is through structured questionnaires. Although the data are participants’ self-reports (primary quantitative data), information is provided through pre-defined categories and aggregated. Thus, information arising from these data requires considerable reconstruction of the individual’s beliefs, actions, *etc.* The question’s meaning is removed from the context of the everyday world and is placed within an empirical context.

Another common way of ‘looking at’ phenomena uses administrative databases (secondary quantitative data). People’s experiences are categorized for specific purposes, often those of government agencies [42], but researchers use this information to answer research questions. In such research, the categories are highly pre-defined, although it is not always obvious (or consistent) how particular experiences are coded into categories. Ambiguous cases may be coded differently by different record keepers. Thus, there is ‘social construction’ even of these statistical ‘facts’. Hence, in an administrative database, phenomena like bicycle injuries are reconstructed artifacts with meaning far removed from the experienced phenomenon. Secondary quantitative data require a degree of reconstruction most distant from the paramount reality.

### 4.3. Complementarity of Perspective

The ‘objective’ and the ‘subjective’ are profoundly different ways of understanding phenomena. However, if these represent the outside and the inside of a box, neither can exist without the other but are mutually dependent and intricately entwined. The division between them is fluid rather than fixed and impervious. The quantitative research paradigm is constructed on what is often referred to as ‘scientific rationality’; however, this scientific rationality is itself constructed on people’s realities [32]. These perspectives explore different dimensions of the same phenomenon. Moreover, they are interdependent, depending on each other for clarity of understanding. Each reality is valuable, yet neither perspective is adequate alone: When these ways of knowing are combined in a complementary manner, the phenomenon under study are understood in terms of both outside generalities and inside particularities, which differ in their levels of reconstruction and their relationships [25].

Furthermore, a complementary integration of these co-existing and inter-related dimensions of phenomena leads us to a more holistic understanding of those phenomena *and* to a more comprehensive view on how phenomena change and evolve. That is, a complementary integration allows us to understand that the causal relationships among the dimensions are reciprocal and that the nature of the ‘whole’ is fluid, rather than static. For example, changes in the frequency of crashes at a particular intersection can engender different perceptions of community safety; and vice versa, changes in individuals’ perceptions of community safety can lead to behavioral changes, affecting the number of crashes that occur. The interdependence of these two paradigms and their synergistic impact on each other are key concepts in Rothe’s mixed methods framework.

## 5. Levels of Reconstruction as Complementarity

When we speak of reconstruction of meaning, we do not mean that the phenomenon itself changes, only its meaning changes. We illustrate this with a hypothetical example, with relevance in injury epidemiology. Imagine that I am distressed, having just been served with divorce papers, and I drive hastily to see my lawyer. In my distracted state, I collide with a slow moving vehicle and sustain whiplash injuries. I go to the hospital where doctors—and a couple of researchers—ask me for information. My ‘lived reality’ may include sadness because of my marital situation; fear and pain related to my whiplash; anger about the collision (perhaps I blame the other driver for driving slowly); frustration at the hospital wait, and so on. At its most immediate level, this reality is directly accessible only to me.

A qualitative researcher wants to enter inside the box and asks me questions about the event. This is the most straightforward way of understanding my experiences, but my reactions to the researcher’s queries might also influence my descriptions. I describe my experiences in detail (and with feeling!) to the researcher, who attempts to analyze, interpret and report this incident and the meaning it has for me. The researcher has not directly experienced the events and does not necessarily understand the experienced reality of my world, so she/he approximates (reconstructs) that experienced reality through the process of inter-subjectivity and shared language. I am given the opportunity to expand and go into depth into issues like my attributions for the crash, my sadness over my loss, my fears for my financial security, my sense of failure, my sense of remorse for having crashed into another car.

As I wait in the emergency room, another researcher asks a series of open-ended questions about my experience. My answers are recorded verbatim, specific phrases are extracted and then sorted into categories. These categories may have been developed a priori based on theory or prior research findings, or may be developed post hoc based on the perceived meaning of pieces of my discourse—an identification of themes. Where the categories (themes) are developed for analysis *on the basis* of my responses, this can be considered a step away from my lived reality, but is still a form of “looking in”. Where my answers are sorted into categories on the basis of *pre-planned groupings* (e.g., whether or not I am experiencing an ‘acute stress reaction’ according to psychiatric classification codes), this falls more within the “looking at” realm, that is, use of a commonly used qualitative way of collecting data for quantitative coding and analysis. Data about my experience are collected and used to expand our understanding about crash sequelae, but are not used to understand the *experienced* reality of the event.

Yet another researcher gives me a questionnaire with items such as a pain scale, a symptom checklist, a question assessing ‘fault’ for the crash, and questionnaire of my feelings. This provides information about my experienced reality at second-hand that can be compared to other such responses, but lacks the richness and the personal meaning of the first-hand experience. As I complete the questionnaire, I attempt to massage my experiences into the structured format of the response options. It will not be an exact fit. For example, I might answer the ‘at fault’ question with “yes”, which is the legally correct answer. However, given my belief that the slow driver was ultimately (but not legally) responsible, my response reflects only part of my reality. In addition, the structured questions do not address the richness of my post-crash feelings or the context of the collision—my distress over an impending divorce. Hence, the survey distorts or reduces the depth and breadth of my injury experience. Although my responses represent my experience, they move outside of the box. Finally, the hospital reconstructs my experience into diagnostic codes that are removed from the meaning of the event. This is the most distant level of reconstruction of the experienced reality, where the feelings, behavior and personal cost become superfluous, the context of the injury is not addressed, and the features of my experience are reduced to an administrative data point—the outside of Bergson’s Box. Yet, these data points can be used in research aimed at developing best practices for emergency room interventions for whiplash injuries or at improving the traffic flow along that particular stretch of highway, thus reducing collisions.

These are brief and simplistic descriptions of what are, in reality, very complex processes of research paradigms and methods. However, our intent in oversimplifying these issues is to demonstrate that at each point, the *meaning* of the event changes. There is no one correct research method, no one correct meaning and this framework is not hierarchical in nature. No particular mode of investigation has more merit. Rather, the modes of inquiry—looking in and looking at—and the paradigms from which these modes of inquiry arise—form a dialectic, unified whole, reflecting a continuum of language and interests. It is by focusing on all the points on the continuum and attending to the specific meaning at each point (being aware of how that meaning changes and evolves) that a comprehensive understanding can be achieved.

Reciprocity among these paradigms is also a critical feature. In the above example, we have a number of different levels of reconstruction of meaning. When understood together, these generate a coherent and comprehensive understanding of crash phenomena, and can inform a broad-based policy designed to reduce crashes and lessen their negative consequences. From the most distant level of reconstruction of meaning, we have coded information on the cause of crash that, when aggregated with coded information from other crashes (as, for example, in a motor vehicle accident database) could lead to policy changes such as better road design or more effective speed limit enforcement. When my responses to the survey are aggregated with other respondents, we have self-report information about the effect of mood and distress on driving. This could lead to policies such as public education programs to increase recognition of the adverse effects of driving while angry or upset. Identifying the incidence of ‘acute stress reaction’ in reaction to a car crash can assist in determining the need for implementing a post-crash mental health monitoring system. An analysis of the qualitative interview can aid in understanding how the crash came to be, why I am so distressed after the crash and—in general—helps us to understand the important contextual factors before, during and after the crash. In short, information gained in qualitative interviews can help us better understand findings from questionnaire research and crash database research, and—vice versa—aggregate findings from questionnaires can determine the frequency of emotions expressed in the interview. The different meanings inherent in each form of research help us formulate a coherent and complementary whole and take actions that reduce injuries.

## 6. Conclusions

The quest for integration of the qualitative and quantitative research paradigms is not easily realized, and a clear path to it needs to be theoretically based and conceptually sound. Each paradigm on its own can be used to expand knowledge and to help in developing policy, as can a complementary integration of these paradigms. However, we must understand the empirical processes, language rules and philosophical assumptions that support the use of mixed methods in research. We aimed to present a framework of *complementarity*—an approach to viewing the integration of qualitative and quantitative methods. The approach must also be sound from the viewpoint of researchers and must also reflect the everyday lives of those who are injured and their caregivers. Use of mixed methods in injury research should progress with a solid foundation of understanding why it is possible to integrate different sources of knowledge and methods of investigations; and, through understanding data construction and reconstruction and attending to different levels of meaning, to explore how injury happens, how risks work in everyday life and how groups of people become vulnerable to injury.
